# Different training responses to eccentric endurance exercise at low and moderate altitudes in pre-diabetic men: a pilot study

**DOI:** 10.1007/s11332-017-0392-3

**Published:** 2017-08-23

**Authors:** Kultida Klarod, Marc Philippe, Hannes Gatterer, Martin Burtscher

**Affiliations:** 1 Department of Sport Science, Medical Section, University of Innsbruck, 6020 Innsbruck, Austria; 2 Department of Physical Therapy, Faculty of Allied Health Sciences, Burapha University, Chonburi, 20131 Thailand; 3 Department of Sports Medicine, Institute of Sports Sciences, Justus-Liebig-University, 35394 Giessen, Germany

**Keywords:** Diabetes, Downhill walking, Oxidative stress, Antioxidants

## Abstract

This pilot study aimed (a) to evaluate the effects of eccentric exercise training at low and moderate altitudes on physical fitness in pre-diabetic men and (b) to establish whether or not oxidative stress levels and antioxidant status were associated with performance improvements. In this crossover trial, five pre-diabetic men conducted nine downhill walking sessions (3 days/week, 3 consecutive weeks) at low altitude (from 1360 to 850 m) and one year later at moderate altitude (from 2447 to 2000 m). Exercise testing and the determination of parameters of oxidative stress and antioxidant capacity were performed pre- and post-training. The biological antioxidant activity of plasma (BAP) increased after eccentric training at moderate altitude (*p* < 0.001), whereas diacron reactive oxygen metabolites (dROMs) remained unchanged. Also, the BAP/dROMs ratio increased only after training at moderate-altitude training (*p* = 0.009). Maximum power output improved after training at low altitude and the changes were significantly related to baseline BAP/dROMs ratio (*r* = 0.90). No decrease was seen for fasting plasma glucose. Eccentric exercise training in pre-diabetic men improved performance only when performed at low altitude and this improvement was positively related to the baseline BAP/dROMs ratio. In contrast, 3 weeks of eccentric exercise training increased BAP levels and the BAP/dROMs ratio only at moderate altitude without improving the performance. Thus, one might speculate that the BAP/dROMs ratio has to increase before performance improvements occur at moderate altitude.

## Introduction

Diabetes mellitus type II is a major public health problem with more than 25 million people in Europe affected by the disease [1]. The disorder results from interactions between genetic, environmental and behavioral factors [2–4]. The latter may be considered a modifiable risk factor and mainly denotes poor health behavior in terms of a high caloric diet and physical inactivity. In view of that, it has been persistently shown that reducing caloric intake and being physically active or performing endurance and strength training on a regular basis to improve physical fitness prevent diabetes mellitus type II development [5] and ameliorate glycemic control [6–8].

Unaccustomed eccentric exercise (muscle lengthening contraction form) is associated with muscle damage inducing reduced glucose transporter type 4 (GLUT4) levels that seems to be the major reason for transient insulin resistance [9–11]. However, it has been shown that repeated bouts of eccentric exercise are protective against further damage caused by subsequent eccentric sessions [12, 13]. Furthermore, it was recently reported that eccentric exercise is not only an effective training method to increase muscle strength and endurance, but may also increase glucose tolerance comparable to concentric exercise [14–20]. Eccentric endurance exercise might be implemented by downhill walking/hiking in a hilly or mountainous environment. In contrast to uphill walking which is considered a concentric endurance exercise, downhill walking opposes less strain on the cardiovascular system but a relatively high strain on the contractile units of muscles [21, 22]. Therefore, downhill walking might be a valuable training modality for persons displaying a low physical fitness and impaired glucose metabolism, as for example subjects suffering from pre-diabetes.

Besides the possibility of mountainous environments to easily perform eccentric endurance exercises, increasing altitude also involves decreasing atmospheric pressure leading to hypobaric hypoxia. While altitudes above 1500 m lead to an altitude-dependent decline of exercise capacity [23, 24], hypoxia has been shown to improve glucose transport and insulin action [25–27] and furthermore may be a stimulus for increasing exercise performance [28]. In this regard, research has shown that both eccentric exercise and hypoxia may induce inflammation and increase reactive oxygen species (ROS) [29–33]. High levels of contraction-induced ROS have been associated with muscle damage and impaired muscle function [34] and acute exercise under hypoxic conditions promotes DNA strand breaks and oxidative DNA damage more than exercise under normoxia [35]. However, contraction-induced ROS also activates important cell signaling pathways mediating training adaptations such as improved antioxidant capacity [36], mitochondrial biogenesis [37–39] and hypertrophy [40]. In fact, it was demonstrated that positive exercise-induced adaptations such as vasodilation [41], mitochondrial biogenesis [39, 42] and insulin sensitivity [43] are blunted when loading cells with high doses of antioxidants or after oral antioxidant treatment. Moreover, exercise-induced ROS production is highly individual and persons producing less exercise-induced oxidative stress exhibited the lowest training adaptations after endurance training [44–46]. It is also known that overweight persons with type II diabetes display higher basal levels of oxidative stress that can be reduced by exercise training, but the link between reduction of basal oxidative stress and improved glucose metabolism has not yet been confirmed [47, 48]. Additionally, eccentric exercise was reported to increase the antioxidative capacity and thus to be protective against oxidative injury [49]. Thus, we assumed that eccentric endurance training performed at low or moderate altitude (i.e., 2000–2500 m) might have distinct effects on redox status and could differently impact on physical fitness.

The effects of eccentric exercise training at moderate altitude on exercise performance and redox status in pre-diabetic people are not established yet. Therefore, this study aimed (a) to evaluate the effects of eccentric exercise training at low and moderate altitudes on physical fitness in pre-diabetic men and (b) to establish whether or not oxidative stress levels and antioxidant status are associated with performance improvements. We hypothesized that eccentric exercise training performed at moderate altitude will improve performance more than at low altitude and that redox changes may at least partly explain the expected differences.

## Methods

### Participants

Five pre-diabetic men (age 56.8 ± 5.8 years; weight 83.8 ± 10.7 kg; height 173.8 ± 3.1 cm) gave their written informed consent to participate in the study. Pre-diabetes was defined according to the American Diabetes Association [50] and included participants with impaired fasting glucose and/or impaired glucose tolerance. Impaired fasting glucose was defined as fasting plasma glucose levels ≥100 and <126 mg/dl, and impaired glucose tolerance was defined as 2-h plasma glucose values in the oral glucose tolerance test of ≥140 and <200 mg/dl. Exclusion criteria were any kind of acute or chronic diseases, smoking and body mass index >30 kg/m^2^ or musculoskeletal problems not compatible with the used downhill walking exercises. The baseline characteristics of the study participants are shown in Table 1. The study was carried out in accordance with the ethical standards laid down in the Declaration of Helsinki and the protocol was approved by the ethics committee of the Medical University of Innsbruck (Protocol ID: AN5029; ClinicalTrials.gov ID: NCT01890876).

**Table 1:** Baseline characteristics of the participants under normoxic (low altitude) and hypoxic (moderate altitude) conditions

Parameters	Normoxia (*N* = 5)	Hypoxia (*N* = 5)	*p* values
Weight (kg)	83.8 ± 10.7	83.5 ± 8.5	0.735
BMI (kg/m^2^)	27.8 ± 3.5	27.6 ± 3.5	0.795
*P*_max_ (W)	161.2 ± 47.6	188.2 ± 47.1	0.007
Hf_max_ (bpm)	145.4 ± 14.9	151.4 ± 13.8	0.260
Fasting plasma glucose (mg/dl)	127.4 ± 39.8	116.4 ± 22.7	0.371
*V*O_2max_ rel. (ml/min/kg)	31.5 ± 10.6	32.3 ± 9.3	0.405

Values are mean ± SD

*Hf*_*max*_ maximum heart rate reached during maximum exercise capacity testing, *P*_*max*_ maximum power output reached during maximum exercise capacity testing, *VO*_*2max*_ *rel.* relative maximum oxygen uptake achieved during exercise capacity testing

### Procedures

The present study is a follow-up of an initial project which was designed as an investigator-initiated two-group random selection pre-test post-test trial. In that trial, we investigated the effects of 3 weeks of uphill and downhill walking at low altitude on fat and glucose metabolism in pre-diabetic men [51]. The eccentric walking protocol has been repeated 1 year later at moderate altitude. Primarily, based on the results of Smutok et al. [52], we calculated a sample size of at least eight participants for the initial project (G*Power, Version 3.1.5). However, of the eight subjects participating in downhill walking in the first year, only five agreed to repeat the downhill walking program at moderate altitude. The low and the moderate altitude training periods were accomplished 1 year apart with the low-altitude training trial performed first. The methodological approach was the same for both periods. Each testing and training period included two pre-test days, nine downhill (eccentric exercise) walking sessions and two post-test days. During the first pre-test, fasting venous blood samples were collected and the participants underwent a routine medical examination and anthropometric measurements. On the second day, the participants performed a symptom-limited incremental exercise test (cycle ergometer test). One week after the pre-tests, the downhill walking training period started. The nine walking sessions were performed on Mondays, Wednesdays and Fridays on three consecutive weeks. The participants were asked to walk as fast as possible or at a maximum intensity corresponding to an individual RPE of 15 [53]. Participants were not allowed to run. The starting point for the low-altitude training period was at an altitude level of 1360 m above sea level and the finish point was at an altitude level of 850 m above sea level. The path had a relatively continuous slope of 10.2% and a length of 5000 m. The mean walking time per session was 69.3 ± 5.1 min. The starting point for the moderate altitude walking sessions was at an altitude level of 2447 m above sea level and the end point was at an altitude level of 2000 m above sea level. The path had a relatively continuous slope of 9.7% with a length of 4600 m. The mean walking time per session was 57.0 ± 3.8 min. During both training periods participants were brought to the starting point by car or cable car. Exercise testing after the training program was conducted on the same day as the last walking session. Fasting blood sampling and anthropometric testing took place 1–2 days after the last walking session.

### Measurements during walking

The heart rate and walking time of each participant were monitored and stored for every walking session by a Polar watch (RS800CX, Polar Electro OY, Kempele, Finland). The average rate of perceived exertion was noted after each walking session. At the beginning of each walking session, delayed onset muscle soreness (DOMS) of the previous walking session was assessed. A graded scale for muscle pain and muscle soreness ranging from 1 to 10 (1 meaning no pain/soreness and 10 meaning maximal pain/soreness) was used similarly as done by Coratella and Bertinato [54].

### Exercise testing

Exercise capacity was assessed by a symptom-limited cycle ergometer test (Excalibur Sport, Lode, The Netherlands). We expected a peak performance of our study participants between 150 and 200 W. Since the recommended duration for incremental exercise tests to exhaustion is generally between 8 and 12 min, we decided to start with an initial load of 50 W for 3 min followed by 50 W increments every 3 min up to exhaustion, resulting in a total test duration of 9–12 min. During the test, gas analyses were performed (Oxycon Alpha, Jaeger, Germany) and the participants were continuously monitored by ECG (Schiller AT-10, Austria). The main outcome parameters were the maximum heart rate, maximum power output and the maximum relative oxygen uptake.

### Fasting plasma glucose

Fasting venous blood samples were taken to quantify fasting plasma glucose using a commercially available enzymatic kit (Roche Diagnostic Systems, Basel, Switzerland) on a Hitachi 902 Autoanalyser (Roche Diagnostic Systems, Basel, Switzerland) at the laboratory of the Medical University of Innsbruck.

### Oxidative stress and antioxidant status markers

Hydroperoxides were measured by the plasma levels of diacron reactive oxygen metabolites (dROMs) using the Free Carpe Diem device (FREE^®^ Carpe Diem; Diacron International, Italy). dROMs levels of plasma in the presence of iron can develop alkoxyl and peroxyl radicals. The radicals can oxidize an alkyl-substituted aromatic amine, thus converting them into a pink-colored derivative. It can be detected by a spectrophotometer at 546 nm. The results are expressed in arbitrary units, namely Carratelli units (U.CARR). A single U.CARR corresponds to 0.08 ng/100 ml of H_2_O_2_ [55].

The biological antioxidant activity of plasma (BAP) was determined using the Free Carpe Diem device (FREE^®^ Carpe Diem; Diacron International, Italy). The test is based on a colored solution containing ferric Fe^3+^ ions bound to a chromogenic substrate which is decolorized on reduction of Fe^3+^ to Fe^2+^ ions by a reducing power of the antioxidant activity of plasma added to the reaction solution. This reaction can measure photometrically the intensity of decoloration at wavelength 505 nm. The normal value for BAP in healthy subjects is >2200 μmol/l [55].

### Statistical analysis

Data analyses were performed using SPSS version 18.0 (SPSS Inc., Chicago, IL, USA). All values are presented as mean ± SD. Low- vs. moderate-altitude training session parameters were evaluated with the use of the Wilcoxon test. Differences between low- and moderate-altitude training outcomes of BAP, dROMs, fasting plasma glucose, maximum power output, maximum heart rate and body mass index were calculated by using two-factor [condition: normoxia (low altitude) vs. hypoxia (moderate altitude) and time: pre- vs. post-training] repeated-measures analysis of variance (ANOVA). Bivariate correlation analyses were performed using Pearson or Spearman’s rank correlation as appropriate. A *p* value ≤0.05 was considered statistically significant.

## Results

### Walking session results

The results of the parameters assessed during walking are presented in Table 2. The mean walking time was significantly lower for moderate-altitude walking compared to low-altitude walking. No other training parameters differed significantly between the two training regimens. Downhill walking at moderate altitude caused no subjective DOMS, while downhill walking at low altitude caused a rather negligible amount of subjective DOMS.

**Table 2:** Results of the nine downhill walking sessions under normoxic (low altitude) and hypoxic (moderate altitude) conditions

	Normoxia (*N* = 5)	Hypoxia (*N* = 5)	*p* values
Mean walking time pws (s)	4159 (304)	3421 (227)	0.043*
Hf_mean_ (bpm)	93.2 (9.8)	98.4 (5.7)	0.138
RPE_mean_ [6–20]	8.9 (1.1)	8.5 (0.4)	0.273
Pain mean [1–10]	1.1 (0.1)	1.0 (0.0)	0.180
Soreness mean [1–10]	1.2 (0.2)	1.0 (0.0)	0.109

Values are as mean ± SD

*pws* per walking session, *Hf*_*mean*_ mean heart frequency, *RPE*_*mean*_ mean rate of perceived exertion

* *p* ≤ 0.05

### Oxidative stress and antioxidant parameters

**Fig. 1:**
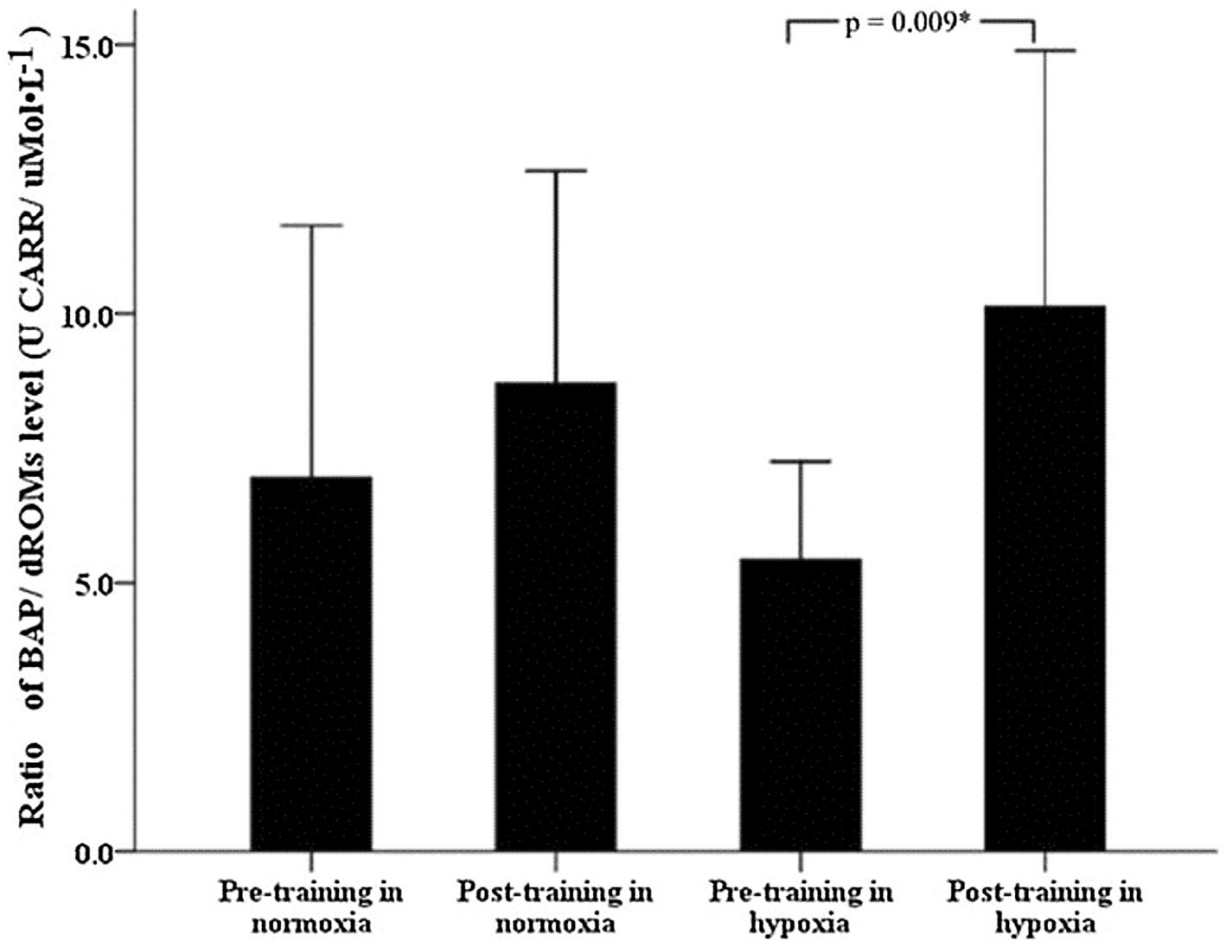
Ratio of BAP/dROMs levels at pre-training and post-training in normoxia and hypoxia groups

Table 3 shows the outcomes of the oxidative stress and antioxidative defense parameters. dROMs levels were neither affected by training (time effect, *p* > 0.05) nor by altitude condition (interaction effect, *p* > 0.05). On the contrary, BAP changed over time with an interaction effect (time × altitude level, *p* < 0.05). Post hoc analyses revealed that BAP levels increased only after eccentric training at moderate altitude (*p* < 0.001). The BAP/dROMs ratio indicating redox balance increased also after training at moderate-altitude training (*p* = 0.009, Fig. 1), but was unaffected at low altitude.

**Table 3:** Pre- and post-training levels of dROMs and BAP under normoxic (low altitude) and hypoxic (moderate altitude) conditions

	Pre-training (mean ± SD)		Post-training (mean ± SD)		Main effect time (*p* values)	Interaction time × condition (*p* values)
	Normoxia	Hypoxia	Normoxia	Hypoxia		
dROMs (UCARR)	234.8 ± 38.7	231.4 ± 31.1	173.8 ± 34.0	231.6 ± 40.8	0.103	0.171
BAP (μmol/l)	1575.6 ± 301.1	1241.2 ± 142.8	1460.5 ± 73.2	2279.5 ± 202.0	<0.001*	0.006*

*dROMs* the diacron reactive oxygen metabolites, *BAP* the biological antioxidant activity of plasma

* *p* ≤ 0.05

### Anthropometric, performance and metabolic data

Table 4 shows the outcomes of the anthropometric, performance and metabolic data. Training and altitude level did not affect body mass index, maximum heart rate and fasting plasma glucose (*p* > 0.05 no time or interaction effects). On the contrary, a significant interaction effect (time × altitude condition) was found for maximum power output which only improved after training at low, but not at moderate altitude.

**Table 4:** Changes of anthropometric, exercise capacity and metabolic parameters under normoxic (low altitude) and hypoxic (moderate altitude) conditions

	Mean differences post minus pre ± SD		Main effect time (*p* values)	Interaction time × condition (*p* values)
	Normoxia	Hypoxia		
Δ BMI (kg/m^2^)	−0.05 ± 0.36	0.12 ± 0.37	0.829	0.231
Δ Hf_max_ (bpm)	2.20 ± 10.11	7.00 ± 9.35	0.226	0.460
Δ *P*_max_ (W)	9.00 ± 8.00	−2.00 ± 10.84	0.436	0.015*
Δ Fasting plasma glucose (mg/dl)	−14.40 ± 29.68	5.00 ± 5.00	0.563	0.176

*Hf*_*max*_ maximum heart rate reached during maximum exercise capacity testing, *P*_*max*_ maximum power output reached during maximum exercise capacity testing

* *p* ≤ 0.05

### Correlations

**Fig. 2:**
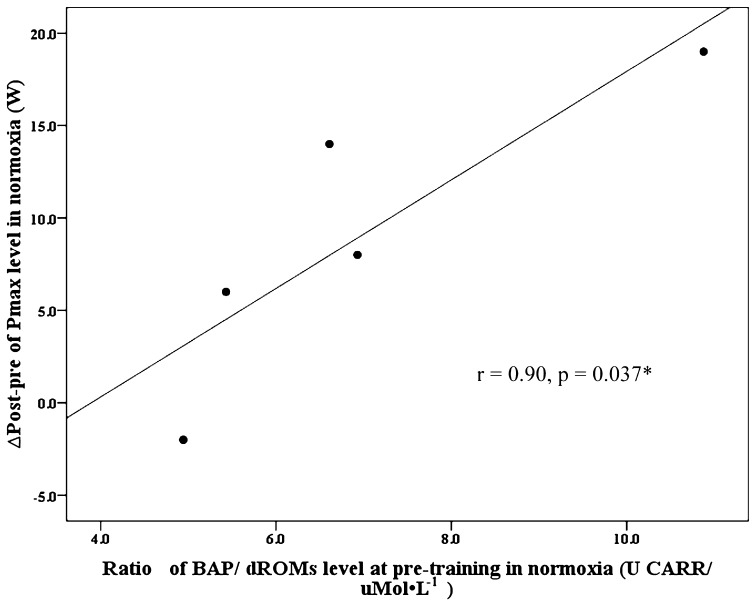
Relationship between the ratio of BAP/dROMs level at pre-training and ∆post–pre of *P*_max_ in normoxia

Correlation analyses revealed that the pre-training BAP/dROMs ratio was significantly related to changes in maximum power output after the low-altitude training period (*r* = 0.90, *p* = 0.037, Fig. 2), but not after the moderate-altitude training period (*r* = 0.30, *p* = 0.624).

## Discussion

The main findings of the present study were (a) that 3 weeks of eccentric exercise in pre-diabetic men at moderate altitude (hypoxia), in contrast to low altitude (normoxia), resulted in a significant increase in BAP and BAP/dROMs ratio, but did not improve performance and (b) that the BAP/dROMs ratio was positively correlated with performance increase at low altitude. No training-dependent changes in fasting plasma glucose were seen after training at low or moderate altitudes.

In contrast to the present study, Pialoux et al. demonstrated that training under hypoxic condition increased oxidative stress (plasma levels of advanced oxidation protein products and malondialdehydes) and weakened antioxidant capacities (ferric-reducing antioxidant power, alpha-tocopherol and beta-carotene) [56]. The authors proposed that the combination of intense running and hypoxia may generate a cumulative oxidative stress [56]. This discrepancy may likely be explained by different types of stressors which depend upon the type of exercise, intensity of exercise training, duration of exercise training and the type and severity of hypoxia [57]. Whereas Pialoux et al. applied intense running in hypoxia (simulated altitude of 3000 m) [56], participants of our study performed downhill walking (eccentric exercise) from about 2500 to 2000 m. Thus, the low energy demand during non-muscle damaging eccentric exercise in rather moderate hypoxia may have generated enhanced BAP levels. This enhancement of antioxidant levels without change in oxidative stress levels in the present study may be considered beneficial adaptation to exercise training in moderate hypoxia (~2000 m) [58]. Such adaptations to chronic exercise training at low altitude have been reported repeatedly after training in general [59], in already trained people [60] and in response to increments in training volume as well [61]. Thus, antioxidant capacity may be enhanced under chronic oxidative stress conditions due to well-designed prolonged exercise training [60, 62]. In moderate hypoxia, low-intensity eccentric exercise training may generate similar effects, but more intense training or more severe hypoxia would potentially increase oxidative stress likely requiring antioxidant supplementation [57].

It has been proposed that the adaptive changes in antioxidant efficacy may depend on the pre-training level and the duration of training [60, 63] and also on training loads [63]. Thus, the lack of alteration of either oxidative stress or antioxidant capacity by chronic low-intensity endurance eccentric exercise under normoxic condition in the present study might well be due to insufficiently high stressors for activation toward protective increase in antioxidants. This is in line with Margaritis et al. [63] and Dernbach et al. [64] who reported no significant changes in oxidative stress and antioxidant efficacy after low-altitude training in already well-trained people [63, 64]. On the other hand, however, endurance eccentric exercise at low altitude was associated with improvement in exercise performance, and participants with high BAP/dROMs ratios benefited more than those with lower ratios. This observation suggests that a higher BAP/dROMs ratio might favor beneficial training effects on performance. Why this was not true under hypoxic conditions might be due to somewhat more pronounced ROS production at moderate altitude requiring even higher BAP/dROMs ratios. Thus, it would make sense to increase first the BAP as observed before performance improvement occurs.

The observation that fasting plasma glucose did not change differently between normoxic and hypoxic conditions is in accordance with previous studies [65–67], which found that fasting plasma glucose was not affected by living at moderate altitude or endurance exercise training over 3 weeks or less [65–67].

The exploratory research design and the small sample size of the present study are the most important limitations to be mentioned. The exploratory design has been applied because there are no studies available that compared the effects of eccentric endurance exercise in pre-diabetic men between low and moderate altitude. The small sample size is at least partly due to logistical problems of very standardized downhill walking for 3 weeks at low and moderate altitudes. Differences between the walking programs may represent further limitations. We tried to choose very similar walking paths for the low- and moderate-altitude training sessions. We managed to have comparable slope gradients, but the total distance of the two paths differed by 400 m. Somewhat longer walking periods indicated a higher training volume at low altitude, while heart rates in contrast to RPE indicated a higher training intensity at moderate altitude. These volume and intensity differences between the two training regimens might also have affected changes of physical performance and redox status. We cannot totally rule out the presence of muscle damage during post-training tests, as we did not measure creatine kinase or other biomarkers of muscle damage. However, subjective DOMS assessment indicated no muscle damage and it also has been reported that repeated bouts of eccentric exercise are protective against further damage caused by subsequent eccentric sessions [12, 13]. Thus, we assume that post-training measurements have not been affected by exercise-induced muscle damage. Maximum power output was somewhat higher before the training at moderate altitude which could have prevented further improvement. However, the decline in maximum power output post-training may indicate a real lack of training effect at moderate altitude. Keeping in mind that this was a crossover study design, the results regarding BAP seem convincing, but with regard to dROMs the small sample size may have led to a type II error.

## Conclusion and practical applications

Eccentric endurance exercise in pre-diabetic men improved performance only when conducted at low altitude and this improvement was positively related to the baseline BAP/dROMs ratio. In contrast, 3 weeks of eccentric exercise training increased BAP levels and the BAP/dROMs ratio only at moderate altitude without improving the performance. Thus, one might speculate that enhancement of BAP before downhill walking at moderate altitudes might favor the beneficial effects on performance. Antioxidant strategies for prophylactic use might include those providing electrons to radicals such as vitamin C and E [68]. If this is right, one might further speculate that antioxidant pre-treatment in pre-diabetic men would result in similar performance increases due to eccentric endurance exercise at moderate altitude as shown at low altitude.

If confirmed in well-controlled studies on larger samples, the presented findings might be of clinical relevance for many patients performing leisure activities in the mountainous areas of the Alps. On the one hand, patients with low exercise tolerance who are unable to walk uphill may benefit from and enjoy the physically less demanding downhill walking. Depending on the antioxidant capacity, eccentric exercise effects may be somewhat different between low and moderate altitude.
